# LZTS1 downregulation confers paclitaxel resistance and is associated with worse prognosis in breast cancer

**DOI:** 10.18632/oncotarget.1630

**Published:** 2013-12-14

**Authors:** Francesca Lovat, Hideshi Ishii, Monica Schiappacassi, Matteo Fassan, Mattia Barbareschi, Enzo Galligioni, Pierluigi Gasparini, Gustavo Baldassarre, Carlo M. Croce, Andrea Vecchione

**Affiliations:** ^1^ Department of Molecular Virology, Immunology and Medical Genetics, Ohio State University Wexner Medical Center and Comprehensive Cancer Center, Columbus, Ohio, USA; ^2^ Department of Frontier Science for Cancer and Chemotherapy Osaka University, Japan; ^3^ Division of Experimental Oncology 2, Centro di Riferimento Oncologico, National Cancer Institute, Aviano, Italy; ^4^ ARC-NET Research Centre, University and Hospital Trust of Verona, Verona, Italy; ^5^ Departments of Pathology and Medical Oncology, Ospedale Santa Chiara, Trento, Italy; ^6^ University of Rome “La Sapienza”, Department of Clinical and Molecular Medicine, Ospedale Santo Andrea, Rome, Italy

**Keywords:** Lzts1/Fez1, breast cancer, taxanes

## Abstract

The Leucine Zipper Tumor Suppressor 1 (LZTS1) is a tumor suppressor gene, located at chromosome 8p22, which is frequently altered in human cancer. In normal tissue, its ubiquitous expression regulates cell mitosis by the stabilization of microtubule networks. LZTS1-deficient mouse embryonic fibroblasts have been shown to have an accelerated mitotic progression, and a higher resistance to taxanes, microtubule-stabilizing drugs.

We investigate the role of Lzts1 in paclitaxel-resistance in breast cancer cells. Downregulation of Lzts1 expression significantly decreases sensitivity to paclitaxel in vitro. We further analyzed Lzts1 expression by immunohistochemistry in 270 primary breast cancer samples and 16 normal breast specimens.

Lzts1 was significantly downregulated in breast cancer samples and its deregulation was associated with a higher incidence of tumor recurrence, and to a worse overall survival. Moreover, Lzts1-negative tumors were associated with unfavorable outcome after taxanes-based therapy.

Thus our data suggest that Lzts1 deregulation is involved in breast cancer and its immunohistochemical evaluation may serve as a prognostic factor for breast cancer therapy

## INTRODUCTION

Breast cancer is the most common cancer and the leading cause of cancer-related death in women worldwide [1]. About 20-30% patients present with metastatic or locally advanced cancer, and other 30% will develop recurrent or metastatic disease [2]. Chemotherapeutic options include serial administration of endocrine, cytotoxic, or biologic therapies. Among the most commonly used cytotoxic drugs for breast cancer are the taxanes; paclitaxel (Taxol) and docetaxel (Taxotere) [3].

Taxanes are potent cytotoxic microtubule-stabilizing agents [4-7], which interact with beta-tubulin and arrest cells at G2/M phase, blocking normal spindle assembly and cell division. They are widely used in the treatment of breast, ovarian, lung, and head and neck cancers [8-11]. In breast cancer patients, paclitaxel offers significant benefits in overall and disease-free survival in metastatic and early-stage cancers [12], and it is also given as neoadjuvant treatment [13]. However, some patients are insensitive to paclitaxel-based therapy and no biomarker is currently available to adequately identify patients that are less likely to be sensible to taxanes treatment, thus preventing unnecessary side effects.

The leucine zipper putative tumor suppressor 1 (LZTS1, previously named FEZ1) gene was identified as a tumor suppressor gene at 8p22 [14]. Lzts1 expression is ubiquitously detected in normal tissues, but it is frequently downregulated or absent in different human cancers [15-21], including breast cancer [22]. LZTS1 deficient mice develop cancers with diverse histogenetic backgrounds [23, 24], suggesting that LZTS1 acts as a major tumor suppressor gene in multiple cell types.

By the functional point of view, Lzts1 inhibits cancer cell growth through the regulation of the mitotic process [25]. Lzts1 controls Cdk1 activity by steadying the Cdc25C phosphatase, a mitotic activator of Cdk1. As a result, Lzts1 maintains high levels of Cdk1 activity preventing chromosomes missegregation at the metaphase plate. LZTS1 deficient mouse embryonic fibroblasts (MEFs) showed decreased Cdk1 during mitosis and accelerated mitotic progression resulting in improper chromosome segregation. Moreover, in the absence of Lzts1, mouse fibroblasts displayed resistance to paclitaxel- and nocodazole-induced M phase arrest [24].

To evaluate the potential use of Lzts1 as a biomarker for the response of breast cancer to paclitaxel treatment, we target endogenous LZTS1 by shRNA in MCF7, MDA-MC231, MDA-MB463, and T-47D breast cancer cells. We further analyzed Lzts1 expression by immunohistochemistry in 270 primary breast cancer samples and 16 normal breast specimens. These data indicate an important role for LZTS1 deregulation in breast cancer and support its immunohistochemical evaluation as a prognostic tool for clinical applications in breast cancer therapy.

## RESULTS

### Lzts1 downregulation decreases sensitivity to paclitaxel in breast cancer cell lines.

We have previously demonstrated that the loss of Lzts1 in MEFs corresponds to an accelerated mitotic progression, and a higher resistance to paclitaxel-induced M phase arrest [24]. By interfering with microtubule function, the taxanes (i.e. paclitaxel and docetaxel) represent an important class of anti-neoplastic agents in breast cancer treatment, and have been incorporated into the management of breast cancer patients in association with anthracyclins and trastuzumab where and when appropriate [26].

**Figure 1:**
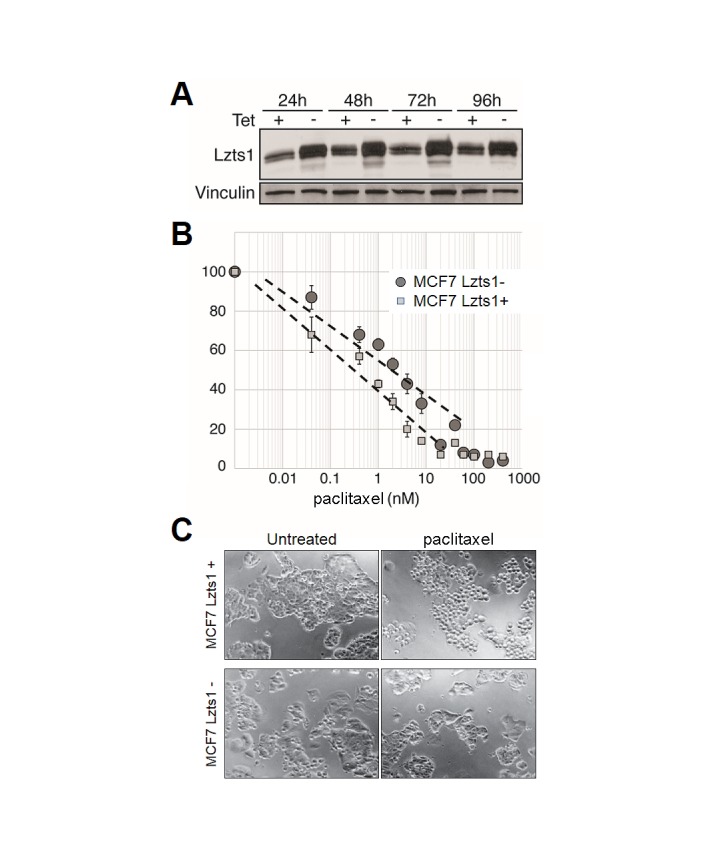
Lzts1 expression sensitizes MCF7 cells to paclitaxel (A) Western blot analysis of Lzts1 expression in Tet-off-inducible MCF7 Lzts1 cells treated or not with tetracycline at different time points. Tetracycline significantly downregulated Lzts1 expression. Protein loading was confirmed by reprobing the membrane with an anti-Vinculin antibody. (B) Dose-response curve after 24 hours exposure to different concentration of paclitaxel in Tet-inducible MCF7 Lzts1 cells treated (Lzts1+) or not (Lzts1-) with tetracycline. Cell viability was plotted against the concentrations of paclitaxel used. Lzts1- cells showed a significantly lower response to treatment until a concentration of 10 nM paclitaxel. Values represent means of triplicate experiments. (C) Representative microphotographs of Tet-inducible MCF7 Lzts1+ or Lzts1- cells treated or not with 2 nM paclitaxel for 24 hours. Lzts1+ cells showed an increased cell rounding and suffering behavior in comparison to Lzts1- clones. (Original magnification, 100x).

To test Lzts1 role in taxanes resistance in breast cancer, we investigate Lzts1 deregulation impact on taxanes sensitivity in four breast cancer cell lines (MCF7, MDA-MB-231, MDA-MB-436, and T-47D). We first used Tet-off-inducible Lzts1 MCF7 clones (Figure 1). Western blot analysis confirmed that the transgene expression was regulated by tetracycline (Figure 1A). The upregulation of Lzts1 was significantly associated to a worse IC50 in the presence of paclitaxel (0.5 nM vs 2 nM; Figures 1B), showing that induced Lzts1 expression increased sensitivity to the therapeutic. Moreover, culture of Lzts1+ transfectants in medium with 2 nM of paclitaxel showed an increased cell rounding and suffering behavior, suggesting that Lzts1 is involved in paclitaxel-induced microtubule stabilization in MCF7 cells (Figure 1C). To further test this hypothesis, inducible MCF7 Lzts1 transfectant cells were exposed to 1 microM Oregon Green paclitaxel (Molecular Probes) for 1 hr and then analyzed by immunofluorescence. Lzts1-induced cells show more distinct and well-organized microtubule network by Oregon Green paclitaxel, respect to Lzts1-uninduced transfectants (Supplementary Figure 1). Overall, these results suggest that Lzts1 loss significantly affects microtubule network, thus reducing microtubule stabilization by paclitaxel.

**Figure 2:**
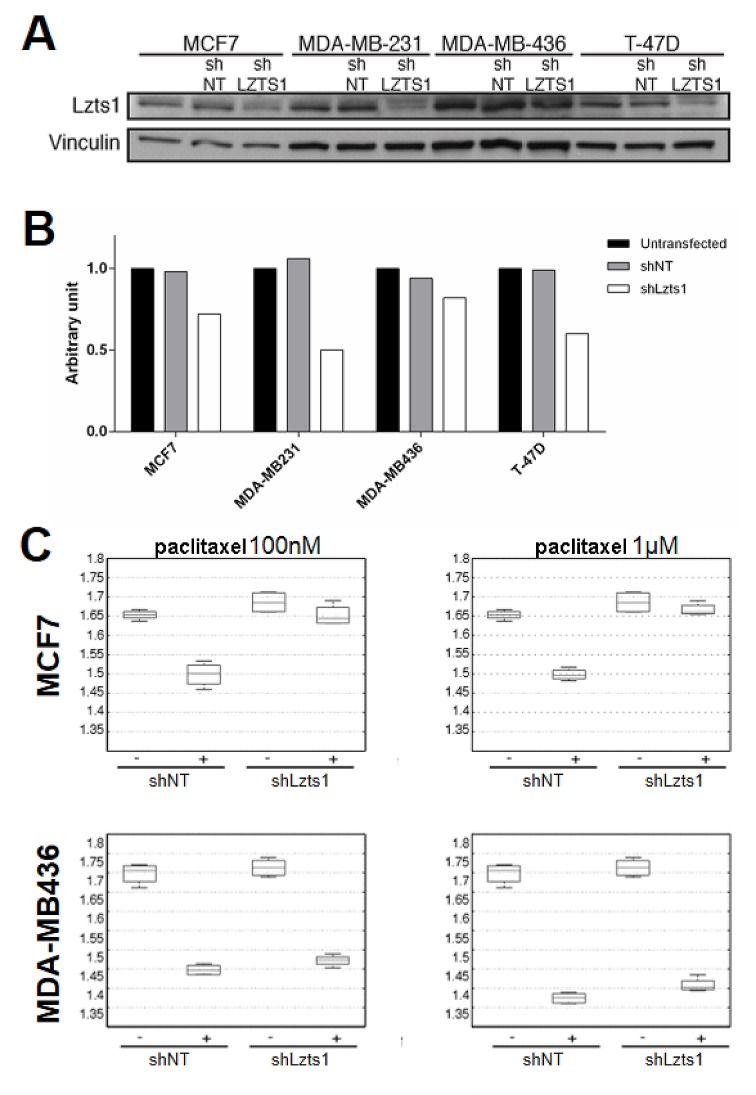
Lzts1 downregulation decreases sensitivity to paclitaxel in breast cancer cell lines (A) Lzts1 expression in breast cancer cell lines transduced with control shRNA (shNT) or LZTS1 shRNAs (shLzts1). Vinculin was used as loading control. Representative image of three separate transductions are shown. shLzts1 led to a significant downregulation of Lzts1 expression in all, but not in the MDA-MB436 cell line. (B) LZTS1 mRNA levels were evaluated by qRTPCR on breast cancer cells after shNT and shLzts1 trasduction. As expected, LZTS1 mRNA was significantly downregulated in shLzts1 MCF7, MDA-MB231, and T-47D clones. (C) Lzts1 expression increases sensitivity to paclitaxel. Box plots show the OD value obtained by reading the MTS plate at 490 nm of MCF7, and MDA-MB-436 cell lines transduced with shNT and shLzts1, treated or not with paclitaxel 100 nM (on the left) and 1 microM (on the right). As expected, shLzts1 MDA-MB436 did not show any significant difference in comparison to the control shNT.

Lzts1 downregulation was established by the use of shRNA (shLzts1) in MCF7, MDA-MB-321, MDA-MD-436, and T-47D human breast cancer cell lines. LZTS1 knockdown was estimated to be 30-50% respect to control shRNA (shNT) in MCF7, MDA-MB-231 and T-47D cell lines by western blot and qRT-PCR analyses (Figures 2A and 2B). MDA-MB-436 cells could not be successfully transduced with anti-LZTS1 shRNA (Figure 2A). In order to evaluate the Lzts1-loss-related paclitaxel resistance, transduced control shNT and shLzts1 cell lines were treated or not with paclitaxel 100 nM and 1 microM and cell viability was tested by MTS assay (Figure 2C and Supplementary Figure 2). As expected, Lzts1 downregulated expression significantly decreased the sensitivity of MCF7, MDA-MB-231 and T-47D cell lines to paclitaxel compared with control (shNT) transduced cells (p < 0.05). Moreover, MDA-MB-436 clones, which showed an unsuccessfully trasduction with anti-LZTS1 shRNA, did not show any difference by paclitaxel treatment among shNT and shLzts1. These data demonstrate that downregulation of Lzts1 protein relatively protects breast cancer cells from the cytotoxic effects of paclitaxel.

### Lzts1 is downregulated and is a prognostic marker in breast cancer

Several recent studies pinpointed a significant downregulation of Lzts1 in human tumors, as well as in breast cancer [22]. To explore the clinico-pathological and therapeutic significance of this association, we investigated Lzts1 expression using the Oncomine database and immunohistochemical analysis in a large series of breast cancers.

**Figure 3:**
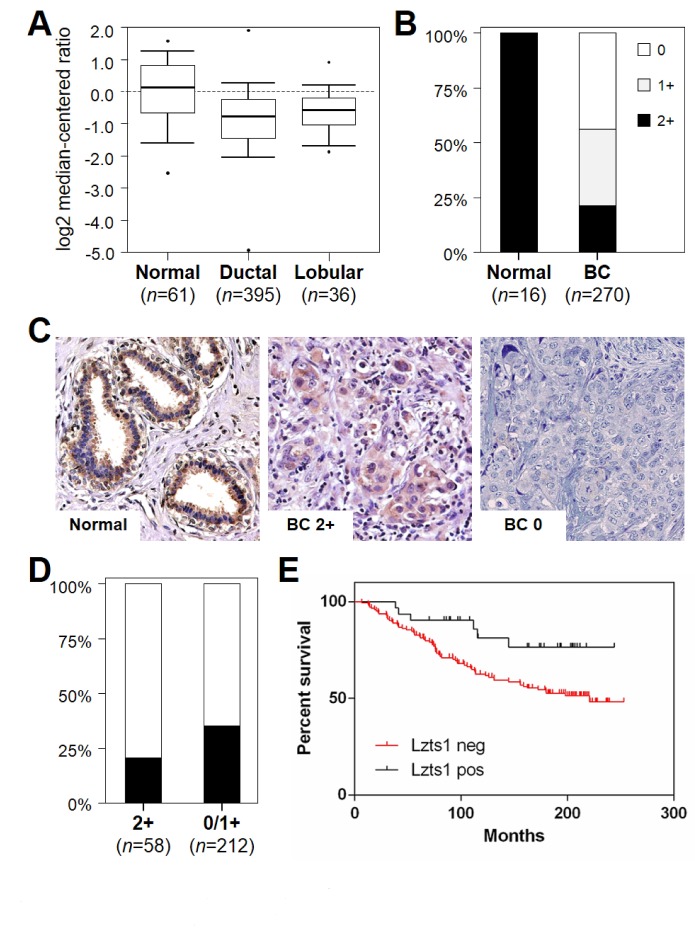
Lzts1 is downregulated in breast cancer and Lzts1 immunohistochemical status inversely correlates with prognosis in breast cancer patients (A) LZTS1 mRNA expression is downregulated in breast cancer. Expression microarray results of the TCGA consortium data set were analyzed, and statistical significance was calculated using the Oncomine website (www.oncomine.org). Box plots show differences in mRNA expression between normal breast, ductal carcinoma, and lobular carcinoma. Data are presented as box plot distribution (line= median value). Numbers represent samples analyzed. (B) Lzts1 immunohistochemical expression is downregulated in breast cancer. Lzts1 staining was significantly lower in cancer specimens than in normal breast samples (p < 0.001). Numbers represent TMA samples available for the analysis. Black= % positive cases, white= % negative cases. (C) Representative images of Lzts1 immunostaining in the normal breast duct, and in two breast cancer specimens. (Original magnification, 200x) (D) Lzts1 immunohistochemical status is associated to tumor recurrence. Lzts1 staining was dichotomized (2+ vs 0/1+). Lzts1 positive cases showed a significantly lower incidence of tumor recurrence (p=0.031). Numbers represent TMA samples available for the analysis. Black= % cases with tumor recurrence, white= % cases without tumor recurrence. (E) Kaplan-Meier analysis of overall survival, according to the expression levels of Lzts1 (2+ vs 0/1+). Patients with strong Lzts1 expression showed a better overall survival in comparison to patients with Lzts1 deregulation (n=175; p=0.022).

The Oncomine database and gene microarray data analysis tool enabled the meta-analysis of gene expression in the breast cancer TCGA microarray studies [27]. The analysis showed a significant LZTS1 mRNA downregulation in invasive ductal (fold change -1.901; p=1.47E-9) and lobular (fold change -1.581; p=1.21E-4) carcinoma samples in comparison to normal breast (Figure 3A).

The Oncomine data were confirmed by immunohistochemical analysis of Lzts1 expression on TMAs. A total of 270 breast cancer specimens were evaluated (Table 1). The series comprised 205 ductal carcinomas and 20 lobular carcinomas (45 cases with different histotype or missing data). All the patients were Caucasian.

**Table 1:** Clinical and demographic characteristics of the breast cancer cohort considered.

Characteristics	Entire cohort	Lzts1 IHC		
		2+	1+	0
Number of cases	270	58 (21.5%)	94 (34.8%)	118 (43.7%)
Age				
mean±SD	59.7±14.0	61.0±14.1	60.0±12.8	58.1±14.9
Histotype				
Ductal	205 (76.0%)	40 (69.0%)	72 (76.6%)	93 (78.8%)
Lobular	20 (7.4%)	3 (5.2%)	8 (8.5%)	9 (7.6%)
Mixed	9 (3.3%)	1 (1.7%)	3 (3.2%)	5 (4.2%)
Others	34 (12.6%)	12 (20.7%)	11 (11.7%)	11 (9.3%)
Missing data	2 (0.7%)	2 (3.4%)	0 (0.0%)	0 (0.0%)
Grade				
I	41 (15.2%)	10 (17.2%)	13 (13.8%)	18 (15.3%)
II	97 (35.9%)	20 (34.5%)	38 (40.4%)	39 (33.1%)
III	124 (45.9%)	23 (39.7%)	41 (43.6%)	60 (50.8%)
Missing data	8 (3.0%)	5 (8.6%)	2 (2.1%)	1 (0.8%)
Death				
No	157 (58.1%)	38 (65.5%)	49 (52.1%)	70 (59.3%)
Yes	113 (41.9%)	20 (34.5%)	45 (47.9%)	48 (40.7%)
Recurrence				
No	184 (68.1%)	46 (79.3%)	58 (61.7%)	80 (67.8%)
Yes	86 (31.9%)	12 (20.7%)	36 (38.3%)	38 (32.2%)
Type of 1st recurrence				
In situ	6 (6.9%)	2 (16.7%)	2 (5.6%)	2 (5.3%)
Local/Regional	20 (23.3%)	4 (33.3%)	8 (22.2%)	8 (21.1%)
Lung	11 (12.8%)	2 (16.7%)	5 (13.9%)	4 (10.5%)
Bone	29 (33.7%)	2 (16.7%)	11 (30.5%)	16 (42.1%)
Liver	10 (11.6%)	0 (0.0%)	6 (16.7%)	4 (10.5%)
SNC	1 (1.2%)	1 (8.3%)	0 (0.0%)	0 (0.0%)
Type unknown	9 (10.5%)	1 (8.3%)	4 (11.1%)	4 (10.5%)
Taxane-based therapies				
No	233 (86.3%)	55 (94.8%)	80 (85.1%)	98 (83.0%)
Yes	37 (13.7%)	3 (5.2%)	14 (14.9%)	20 (17.0%)

Lzts1 was significantly downregulated in breast cancer samples (p<0.001; Figure 3B). A total of 58 tumors (25.1%) showed a moderate/strong Lzts1 expression, comparable to that of the normal counterpart. No significant differences were observed in Lzts1 staining distribution according tumor histotype and grading. Representative examples of Lzts1 immunostaining in breast cancer normal and tumor tissue are shown in Figure 3C.

Lzts1 deregulation (0/1+ cases) was associated to a higher incidence of tumor recurrence (p=0.039; Figure 3D) and to a worse overall survival (p=0.022; Figure 3E). Since Lzts1 expression could affect the result of paclitaxel treatment on different breast cell lines, we investigated associations between Lzts1 expression levels and survival stratifying patients between treated or not with taxan-based chemotherapy. Only in patients with absent Lzts1 expression (n=118), Kaplan-Meier curves showed a clear and significantly trend to more unfavorable outcome after taxan-based therapy (n=20; p=0.031). The low number of Lzts1 2+ taxanes-treated patients did not consent further investigations.

Furthermore to validate these study findings, we confirm the deregulated LZTS1 expression on a different patients cohort using the database published by Esserman et al. (GEO: GSE22226) [28]. From the analysis of this database, based on Agilent Human oligonucleotide microarrays, we verify that patients who are non responders to benefit from taxan-based chemotherapy (n=62) showed a downregulation of LZTS1 expression compared to patients who are responders to taxan-based treatment (n=24) (Supplementary Figure 3).

## DISCUSSION

Taxanes have been successfully introduced in the polychemotherapy regiments for breast cancer treatment, conferring an overall increased patients' survival. However, they are linked to a range of toxicities [29], and thus the identification of predictive biomarkers of tumor responsiveness is required. Data from two major taxane trials [30, 31] provide evidence that ER and HER2 status may aid the selection of patients benefiting from taxane therapy. Nevertheless, no suitable biomarker has been introduced into clinical practice so far.

In previous studies, we have identified LZTS1, as a tumor suppressor gene mapping on chromosome 8p22, whose expression is altered in different human malignancies, including breast cancer. Moreover, we demonstrated that Lzts1 is ubiquitously expressed in normal tissues and it regulates cell mitosis by the stabilization of microtubule networks. In Lzts1-deficient mouse embryonic fibroblasts, the mitotic progression is accelerated and the cells are characterized by a higher resistance to taxanes [24].

In this study, we investigated Lzts1 role in taxanes-resistance in breast cancer, targeting endogenous LZTS1 by shRNA in MCF7, MDA-MC231, MDA-MB463, and T-47D breast cancer cells. As described in MEFs, downregulation of Lzts1 expression significantly decreases the sensitivity to paclitaxel in vitro. Ishii et al. [25] demonstrated that Lzts1 is involved in microtubule assembly and its C-terminal portion is in association with microtubules. These data suggest a correlation between Lzts1 deregulated expression and microtubule-targeting chemotherapeutic sensitivity in cancer cells. We have further characterized Lzts1 as a putative protein responsible for taxanes resistance in breast cancer cells by affecting microtubule network. Lzts1 loss in MCF7 inducible clones leads to a reduced microtubule stabilization and organization, which is usually observed after paclitaxel administration. A similar effect was observed by LZTS1 targeting by shRNA technology. Knockdown of endogenous LZTS1 caused resistance to paclitaxel in MCF7, MDA-MB231 and T47D breast cancer cells, but not in MDA-MB436 where Lzts1 expression remained unchanged. A possible explanation for MDA-MB436 ineffective transduction is that most transduced cells likely die by apoptosis or mitotic catastrophe for an improper cell division. In fact, microtubular poisons, such as taxanes, lead to mitotic catastrophe by binding to beta-tubulin and disrupting the mitotic spindle [7]. Overall these results confirm that the asbence/reduction of Lzts1 expression impairs paclitaxel capability to interfere with the normal disassembly of microtubules during cell division.

In our seminal report [24], we described a significant loss of Lzts1 expression in breast cancer samples and cell lines. In recent years, Lzts1 has been shown to be associated to metastatic disease and a worse patients overall survival [32]. In this study, Lzts1 expression was tested in a large series of breast cancer cases. As previously described, the Oncomine database pinpointed a significant downregulation of LZTS1 mRNA expression in breast cancer samples (p<0.001), which was further confirmed by immunohistochemistry in a series of 270 breast cancers and 16 normal breast specimens. Lzts1 was significantly downregulated in breast cancer (p<0.001), and its deregulation was associated to a higher incidence of tumor recurrence (p=0.039) and of bone marrow metastases (42.1% vs 16.7%). Lzts1 loss was also associated to a worse overall survival (p=0.022). Of interest, absent/low Lzts1 was significantly associated to an unfavorable outcome after taxanes therapy (p=0.031). These findings should be further confirmed in larger series of taxanes-based clinical trials.

These results suggest that Lzts1 plays a critical role in the resistance to paclitaxel, and may potentially serve as a therapeutic target for overcoming paclitaxel resistance in human breast cancer patients. Lzts1 immunohistochemical evaluation could be useful as a prognostic tool for clinical applications in breast cancer therapy.

## MATERIALS AND METHODS

### CDNA microarray analysis

The Oncomine database and gene microarray analysis tool, a repository for published cDNA microarray data (www.oncomine.org) [33] was explored (1st August 2013) for LZTS1 mRNA expression in the TCGA breast cancer series. Oncomine algorithms were used for the statistical analysis of the differences in LZTS1 mRNA expression.

### Patients

A series of 270 consecutive female breast cancer patients were selected from the electronic archives of the surgical Pathology Unit at Santa Chiara Hospital (Trento), and considered for the immunohistochemistry study. Duplicate TMA blocks that each contained single 0.2-mm cores sampled from representative paraffin blocks from each patient were constructed. Patient tumor paraffin blocks were assigned an anonymous unique identifier linked to databases that contained pathological, and clinical data. Estrogen receptor (ER), progesterone receptor (PR), and HER2 (both immunohistochemical and FISH) status were retrospectively obtained from the original pathological reports. Tissues of patients who received adjuvant and/or neoadjuvant chemotherapy were included in the analysis. The clinical and pathologic characteristics of the considered series are summarized in Table 1. A total of 16 normal breast samples obtained from reduction mammoplasty were also included. Institutional review board approval was obtained for the use of patient blocks and the institute's ethical regulations concerning research on human tissues were followed.

### Immunohistochemistry

Immunohistochemical stainings for Lzts1 (Amersham Pharmacia) were obtained on 4 microm-thick sections and performed automatically (Dako Autostainer immunostaining system; Dako), according to the manufacturer's instructions. IHC sections were lightly counterstained with hematoxylin. Appropriate positive and negative controls were run concurrently.

Lzts1 cytoplasmic expression was jointly scored by two pathologists (MB and AV) unaware of any clinical information. Lzts1 staining was classified on a three-tiered scale based on the rate of positive tumor cells: 0, staining in 0-5% of cells; 1+, staining in 5-80% of cells; 2+, staining in >80% of cells. In negative cancer samples, Lzts1 expression in stromal/inflammatory cells and in coexisting non-neoplastic epithelia (if any) served as a positive internal control.

### Cell Culture

Human breast cancer derived cell lines MCF7, MDA-MB-231, T-47D, and MDA-MB-436 were obtained from American Type Culture Collection (ATCC) and maintained in Dulbecco's Modified Eagle's Medium (DMEM), supplemented with 2mM L-glutamine, 100 IU/ml penicillin-streptomycin, and 10% FBS (Sigma).

Gene silencing of human LZTS1 was achieved by shRNA strategies using lentiviral based sh constructs (Invitrogen). For lentiviral production, 293FT cells (Invitrogen) were co-transfected, using Lipofectamine 2000 (Invitrogen) with the lentiviral based sh constructs and lentiviral system vectors pLP1, pLP2 and pVSV-G (Invitrogen). 48 to 72 hours after transfection, medium containing viral particles was used to transduce breast cancer cell lines.

### Preparation of cell lysate and immunoblotting

Cell lysates were prepared using cold NP-40 lysis buffer plus a protease inhibitor cocktail (Complete, Roche), 1 mM sodium orthovanadate, and 1mM dithiothreitiol. Equivalent amounts of protein were loaded on a Criterion Tris-HCl 4-20% pre-cast gel (Bio-Rad) and transferred on nitrocellulose membranes. Anti-Lzts1 (GeneTex Inc) and anti-Vinculin (Sigma) were used as primary antibodies. Isotype-matched, horseradish-peroxidase-conjugated secondary antibodies (GE Healthcare) were used, followed by chemiluminescence detection (Denville Scientific Inc).

### Cell viability assay

Cell viability was detected using the CellTiter 96 Aqueous non-radioactive cell proliferation assay (Promega), following supplier's protocol. A reading of OD 490 nm absorption with a Multilabel Counter (Bio-Rad Laboratories) in a 96-well plate showed linear compatibility with the number of cells in a range between 2000 and 5000 cells/well, counted by trypan blue staining to exclude dead cells. Three independent assays were performed for each experimental group.

### Reverse transcription and quantitative real time PCR (qRT-PCT)

Total RNA was isolated using TRIZOL reagent solution (Invitrogen) and reverse-transcribed using random hexanucleotides as primers and MultiScribe reverse transcriptase (Applied Biosystem). Quantitative real time-PCR was performed with the SYBR Green PCR Master Mix (Applied Biosystems) under the following conditions: 10 min at 95 °C followed by 40 cycles (15 s at 95 °C and 1 min at 60 °C). Glucose-6-phosphate dehydrogenase (GAPDH) was included as housekeeping gene control to correct for equal RNA amounts. Primer sequences and respective probes were designed using the Universal ProbeLibrary (Roche) and the ProbeFinder software (http://www.roche-applied-science.com/): human LZTS1 forward 5'-GAG CCT CAT GAA GGA GCA GG-3', human LZTS1 reverse 5'- CAG GTC CTG GGT CCT CAG CT 3'. All the reactions were run in triplicate, including no-template controls.

### Statistical Analysis

Not all marker or clinical data were available on all subjects, and percentages refer to cases for which data for a specific variable were available. Associations between categorical variables were evaluated using chi-square, t-test, or Fisher exact tests.

Kaplan–Meier and Cox regression models were used to evaluate overall survival (OS), where differences in distributions were evaluated based on clinical characteristics and marker expression. Only patients <75 years of age and with ductal and/or lobular histotype were considered (n=175). The P-values reported in relation to patient survival correspond to log rank tests unless otherwise noted.

## SUPPLEMENTARY FIGURES


