# Supplementary material for: LZTS1 downregulation confers paclitaxel resistance and is associated with worse prognosis in breast cancer
Source: Oncotarget. 2013 Dec 14;5(4):970–7. doi: 10.18632/oncotarget.1630 (PMC4011598; doi:10.18632/oncotarget.1630)
Supplement: Supplementary file 1 [file oncotarget-05-970-s001.pdf]

LZTS1 downregulation confers paclitaxel resistance and is associated with worse prognosis in breast cancer - Lovat et al

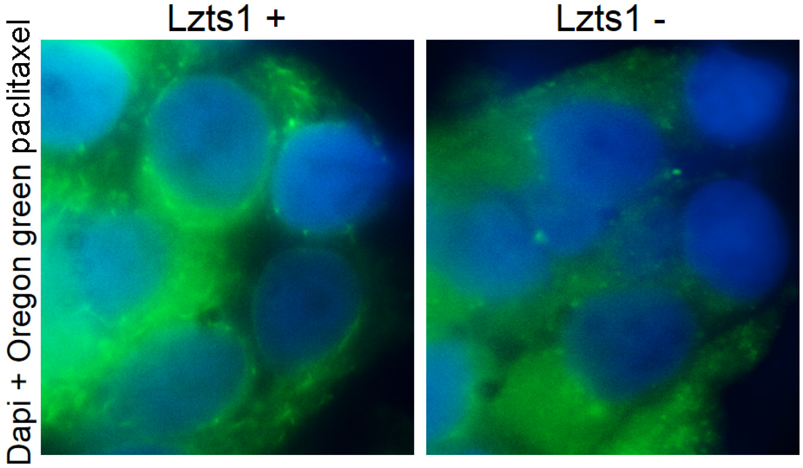

Supplementary Figure 1: Lzts1 downregulation affects microtubule network. Immunofluorescence analysis of Tet-inducible MCF7 cells treated or not with 1microM Oregon Green paclitaxel for 1 hr. Lzts1- clones lack of an evident microtubule network as observed in Lzts1+ cells. Blue, DAPI nuclear staining. (Original magnification, 400x)

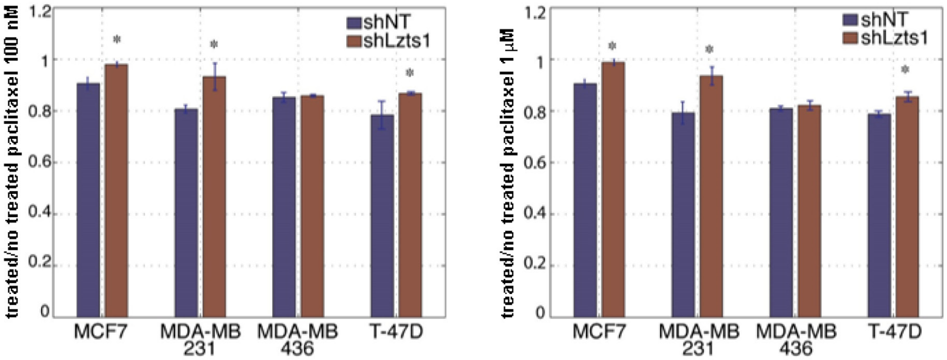

Supplementary Figure 2. Lzts1 downregulation decreases sensitivity to paclitaxel.

Supplementary Figure 2. Lzts1 downregulation decreases sensitivity to paclitaxel. MTS assay performed on breast cancer cells incubated with Taxol 100 nM or 1 microM. Cell viability is represented as the OD value obtained by reading the plate at 490 nm. The experiments were conducted in triplicate and presented as average ± s.d. The blue bars indicate the ratio between the control shNT transduced cells treated and not treated with paclitaxel. The red bars indicate the ratio between shLzts1 transduced cells treated and no treated with paclitaxel. shLzts1 MCF7, MDA-MB231, and T-47D clones showed a significantly lower response to paclitaxel in comparison to control vector (shNT). As expected, shLzts1 MDA-MB436, which showed an ineffective LZTS1 small RNA interference, had similar results to the control shRNA.

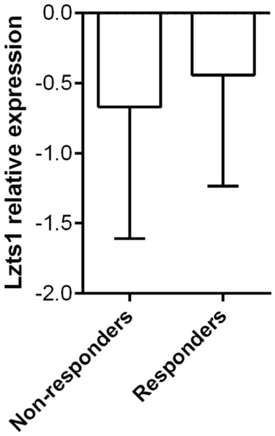

Supplementary Figure 3: Validation of Lzts1 downregulation in non responder patients treated with taxanes. The Oncomine database and gene microarray analysis tool, a repository for published cDNA microarray data (www.oncomine.org) was explored (20th August 2013) for LZTS1 mRNA expression in the Esserman et al. (GEO: GSE22226) 29 breast cancer series. Oncomine algorithms were used for the statistical analysis of the differences in LZTS1 mRNA expression. Box plots show differences in mRNA expression between non responder (n=62) and responder (n=24) patients to taxan-based theraphy.
